# Reconstruction of the orbital floor using supercritical CO_2_ decellularized porcine bone graft

**DOI:** 10.7150/ijms.64359

**Published:** 2021-09-13

**Authors:** Chao-Hsin Huang, Dar-Jen Hsieh, Yi-Chia Wu, Ko‐Chung Yen, Periasamy Srinivasan, Hsiao-Chen Lee, Ying-Che Chen, Su-Shin Lee

**Affiliations:** 1 School of Post Baccalaureate Medicine, Kaohsiung Medical University, Kaohsiung, Taiwan.; 2 Center of Research and Development, ACRO Biomedical Co., Ltd. Kaohsiung, Taiwan.; 3 Division of Plastic Surgery, Department of Surgery, Kaohsiung Medical University Hospital, Kaohsiung, Taiwan.; 4 Regenerative medicine and cell therapy research center, Kaohsiung Medical University, Kaohsiung, Taiwan.; 5 Department of Surgery, Faculty of Medicine, College of Medicine, Kaohsiung Medical University, Kaohsiung, Taiwan.; 6 Department of Surgery, Kaohsiung Municipal Siaogang Hospital, Kaohsiung, Taiwan.

**Keywords:** orbital wall reconstruction, ABCcolla^®^ collagen bone graft, supercritical carbon dioxide, bone graft, xenogenic graft

## Abstract

Orbital floor fractures subsequently lead to consequences such as diplopia and enophthalmos. The graft materials used in orbital floor fractures varied from autografts to alloplastic grafts, which possess certain limitations. In the present study, a novel porcine bone matrix decellularized by supercritical CO2 (scCO2), ABCcolla® Collagen Bone Graft, was used for the reconstruction of the orbital framework. The study was approved by the institutional review board (IRB) of Kaohsiung Medical University Chung-Ho Memorial Hospital (KMUH). Ten cases underwent orbital floor reconstruction in KMUH in 2019. The orbital defects were fixed by the implantation of the ABCcolla® Collagen Bone Graft. Nine out of ten cases used 1 piece of customized ABCcolla® Collagen Bone Graft in each defect. The other case used 2 pieces of customized ABCcolla® Collagen Bone Graft in one defect area due to the curved outline of the defect. In the outpatient clinic, all 10 cases showed improvement of enophthalmos on CT (computerized tomography) at week 8 follow-up. No replacement of implants was needed during follow-ups. To conclude, ABCcolla® Collagen Bone Graft proved to be safe and effective in the reconstruction of the orbital floor with high accessibility, high stability, good biocompatibility, low infection rate and low complication rate.

## Introduction

Orbital fracture is usually the result of a traumatic accident and high-energy injuries lead to impaired alignment and integrity of facial bones, with the symptoms of periorbital edema, tissue lacerations, enophthalmos and diplopia 1-6. Thus, restoring symmetry of orbital walls is critical to improving clinical outcomes 1-3, 5, 6. Various implants ranging from autogenous grafts to alloplastic materials are developed to reconstruct the impaired orbital structure 7. The common implants used in the orbital fractures are autologous cartilage, bone implants, Medpor^®^ (porous polyethylene), and titanium mesh 3, 8, 9. Titanium mesh and autogenous bone grafts were preferred for bridging large defects based on their tough mechanical strength. Titanium mesh implicates osteointegration, but its sharp edge after trimming often causes secondary injury to periorbital soft tissue and induces fibrotic reaction 10-12.

The suitable graft material for the small orbital defect is Medpor^®^ with a curved orientation, malleable and resorbable material 5, 9, 11. Medpor^®^ overcomes most of the advantages of using autogenous graft and titanium mesh. However, Medpor^®^ as alloplastic materials, still exhibit risks of infection and immune rejection 2, 3, 10, 12. Moreover, the cost of Medpor^®^ is unneglectable and can be a burden to patients 3. Autogenous grafts have their limitations such as limited bone availability and increased donor site morbidity 3, 9, 12.

ABCcolla^®^ Collagen Bone Graft, a decellularized porcine bone matrix was used in the reconstruction of the orbital floor. It is a porcine cortical bone matrix processed by supercritical carbon dioxide (scCO2) extraction technology to remove cells, fats, hydrocarbons and non-collagenous proteins while preserving the collagen and bone matrix scaffold structure intact 4, 13, 14. scCO_2_ extraction technology-mediated decellularization process improved the regenerative nature of the xenogenic graft 15. The advantages of using carbon dioxide as a solvent in the scCO_2_ technique are natural, safe, non‐toxic, non‐corrosive, non‐flammable, easily accessible and cost-effective 16. In addition, the scCO_2_ process leaves no chemical solvent residue or off-odours and thus, is relatively more environmental friendly. The scCO_2_ process also effectively remove immunogens and potential pathogens from the animal derived bone materials without compromizing the mechanical strength of the bone matrices 17.

To attain good functional and aesthetic result it is essential to reconstruct the anatomical structure of the orbit, against herniation forces. Natural and synthetic materials are used to reconstruct the orbital walls. However, the choice of the reconstructed material depends on the surgeon's expertise, availability of the material, and drawbacks of using different graft materials. Therefore, we report the cases of orbital floor fracture and reconstruction by using ABCcolla^®^ Collagen Bone Graft. This study aims to introduce a novel means that can be cost-effective and at the same time maintains low complication rate, high resistance to infection and high delicacy of operation.

## Materials and methods

### Patients

Ten cases of orbital wall fractures between the age of 30 to 65 were included in this retrospective study (Table 1). The medical records of these cases were reviewed. Eight cases have resulted from traffic accidents and 2 cases were due to accidental fall. The patients underwent facial bone open reduction and internal fixation (ORIF) surgery in KMUH (Kaohsiung Medical University Hospital) between August 9, 2019, to October 21, 2019. Informed consent from each participant was obtained. In addition, this study was approved by the Institutional Review Board of Kaohsiung Medical University, Kaohsiung, Taiwan (IRB No. KMUHIRB-F(I)-20190044). In addition, this study was performed in accordance with the principles of the Declaration of Helsinki. This case report focuses on the clinical outcomes of orbital wall reconstruction using customized ABCcolla^®^ Collagen Bone Graft, a decellularized xenogenic bone matrix in patients with orbital wall deformities.

### Production of ABCcolla^®^ Collagen Bone Graft

ABCcolla^®^ Collagen Bone Graft was manufactured using supercritical carbon dioxide technology and characterized following Chen et al. 4, 13, 14.

### CT scan

Computerized tomography (CT) by a 16-channel multidetector-row CT scanner (Lightspeed 16; General Electric Medical Systems, Milwaukee, USA) was performed in this study 5. Each CT image was done via a narrow field technique with 3-mm slice thickness 5. CT was used in each case for evaluating the alignment of facial bones and creating a three-dimensional reconstruction image. In this study, coronal and axial projections of CT images were specifically used to identify the sites and extent of orbital bone fracture, as well as the degree of extraocular muscle laceration and periorbital tissue injuries. The quantification of the optical wall framework was done via postoperative CT images. A three-dimensional (3D) reconstruction was created based on CT images to facilitate surgeons to customize implants 18.

### Team of Ophthalmologist

To diagnose the ophthalmologic injuries, patients' clinical symptoms and medical records were reviewed by a team of ophthalmologists. In addition, conducted optical examinations after the surgery and followed up for 4-months. The test data included a primary ocular examination, extraocular movement examination, Hess screen test, and diplopia field test.

### Navigator assisted mirroring tool

**Figure 1:**
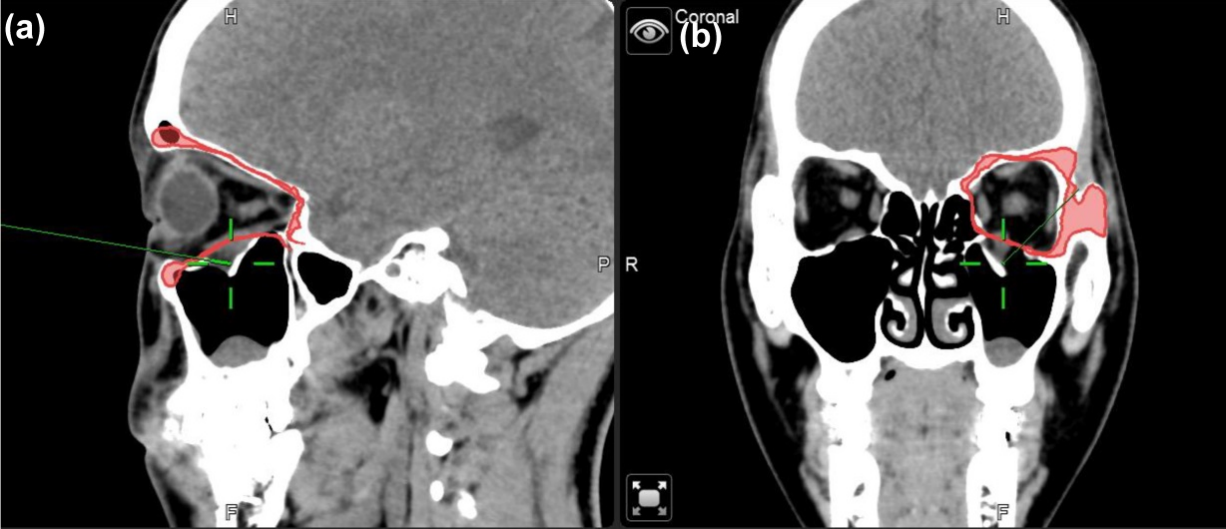
Case 1 30 y/o female patient with left side orbital floor blow out a fracture. Illustration of mirroring and superimposed the unaffected side (right side) object on the affected side (left side) by Brainlab® navigator software tool. The red line indicated the outline of the mirrored object. (a) Sagittal view of preoperative CT image. Greenline pointed at the fractured left orbital floor. (b) coronal view of preoperative CT image. Greenline pointed at the affected left orbital floor.

To minimize the risk of optic nerve injury and variability of clinical outcomes, navigator assisted mirroring tool was used. Mirroring software from the Brainlab navigator system provided precise recreation of orbital wall symmetry before operation. Contouring of unaffected side of the orbital framework was recognized on CT coronal and axial projections. The mirroring tool of the software copied the outline of unaffected orbital walls. Mirroring and superimposed the object to the affected side to simulate the ideal orbital wall position (Figure 1). Plastic surgeons can plan out surgical procedures based on the superimposed images accordingly.

### Surgical procedures

**Figure 2:**
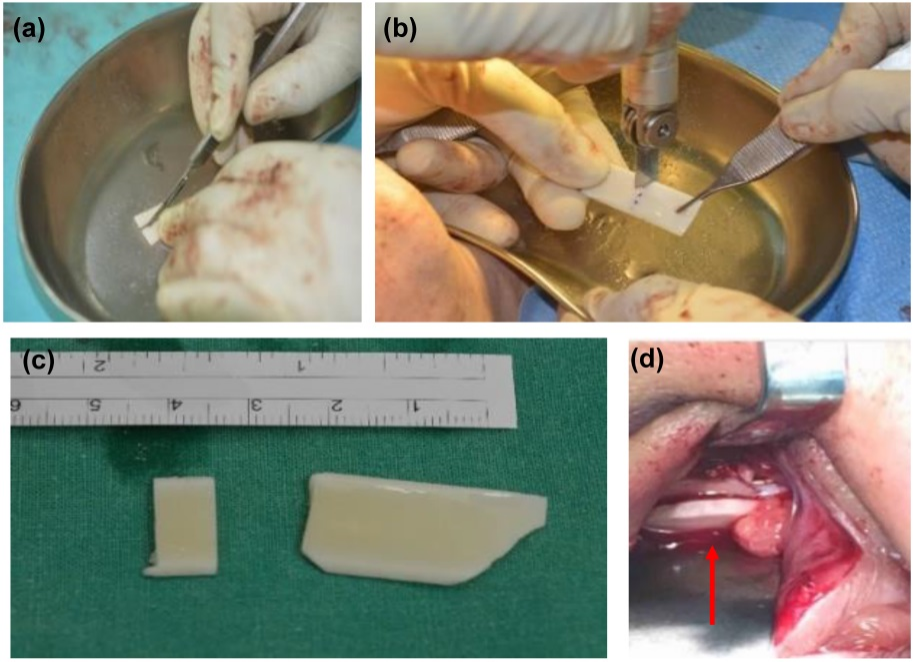
Illustration of customizing ABCcolla Collagen Bone Graft based on the size of orbital wall defect. After the implant was customized, it was placed over the orbital wall defect area as a framework. (a) cutting the implant into a suitable size. (b) customized the shape of the implant according to the shape of the orbital defect. (c) customized implant before placing it into the orbital wall. (d) customized implant after placing into the orbital wall. Size and shape are aligned with the orbital wall.

Ten cases were all operated under general anaesthesia. Nine out of ten cases were performed via the sub-ciliary approach to explore the orbital floor, and one case was performed via the transconjunctival approach. These two approaches had different incision sites to start on 6, 19. The sub-ciliary approach was made first via injection of 2.5ml 2% lidocaine with 1:200,000 epinephrine in a 25-gauge needle into the sub-ciliary area. A skin incision was made laterally following a skin crease from punctum to lateral canthus in the affected side 19. Pre-septal orbicularis oculi fibers were then dissected and separated from the tarsal plate. On the other hand, the transconjunctival approach was performed first also by injecting 0.5 ml 2% lidocaine, with epinephrine (1:200,000) with a 25-gauge needle into the medial bulbar conjunctiva to maintain hemostasis. A forced duction test was then conducted to assess the passive mobility of the affected globe. A 6-0 nylon stitch was made in the middle of the lower eyelid for a stable downward traction force. The transconjunctival incision was made 10-12 mm. After exposing the affected site, a sub-periosteal dissection was performed. Then, the herniated or incarcerated periorbital tissue was identified and reduced using a malleable retractor. Two curved elevators were inserted into the orbital socket and pulled up periosteum and surrounding soft tissue to prevent any tissue entrapment during insertion of ABCcolla^®^ Collagen Bone Graft. The graft was pruned for orbital floor reconstruction. To achieve the desired shape of the graft, a size 15 surgical blade was used to draw the outline on ABCcolla^®^ Collagen Bone Graft. An Oscillating saw was then used to trim the graft into a precise shape. The strips of the bone graft were pruned and built to form a barrel-shaped structure that offers good flexibility for accurate orbital wall reconstruction. Identifying the area of defect was crucial for customizing ABCcolla^®^ Collagen Bone Graft. The customized graft then was inserted into the space created between two curved elevators (Figure 2). To reinforce the stability of the implant, the bone graft was fixed with one 1.0 mm micro screws into the orbital floor. The curved elevator was released after securing the screw to the orbital floor, laying down periosteum and soft tissue to cover the implant naturally. Another forced duction test was done after the operation to check the iatrogenic injury. The periosteum was sutured with 4/0 Vicryl stitches and the wound was repaired using 7-0 Vincryl stitches. Some of the surrounding soft tissue was trimmed to relieve extraocular muscle if extraocular muscle entrapment was observed during the surgery. Using pre-operative CT scan, virtual 3D reconstruction model, navigator assisted mirroring tool, and teamwork with ophthalmologists, operations in this study could precisely implant the xenogenic decellular framework into an accurate position.

### Ethical statement

The study was approved by the institutional review board (IRB) Kaohsiung Medical University Hospital (KMUHIRB-F(1)-20190044). Informed consents were also obtained.

## Results

ABCcolla^®^ Collagen Bone Graft was manufactured using supercritical carbon dioxide technology and characterized following Chen et al. 4, 13, 14.

All of the 10 cases underwent orbital floor reconstruction in 2019 in our department by two senior surgeons. Our institute was one of the major referred medical centers in Kaohsiung city with more than two hundred craniofacial ORIF surgeries carried out annually in average for the past 20 years. Three patients with pure orbital wall/floor fracture were operated under navigator assisted surgery. The others associated with zygomatic complex fracture were operated under conventional technique.

**Figure 3:**
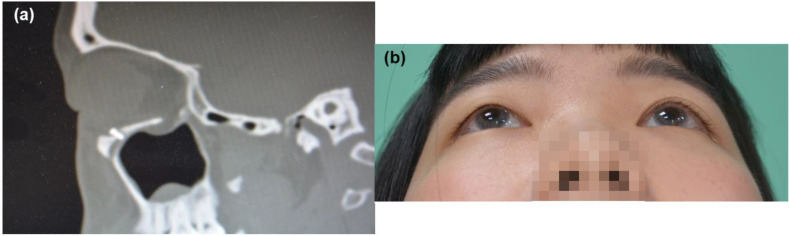
(a) Postoperative CT scan of case 1, left side orbital floor to blow out a fracture. ABCcolla® Collagen Bone graft was fixed by one screw (b) post-operation 2 months follow-up. Without any complaint was noted.

**Figure 4:**
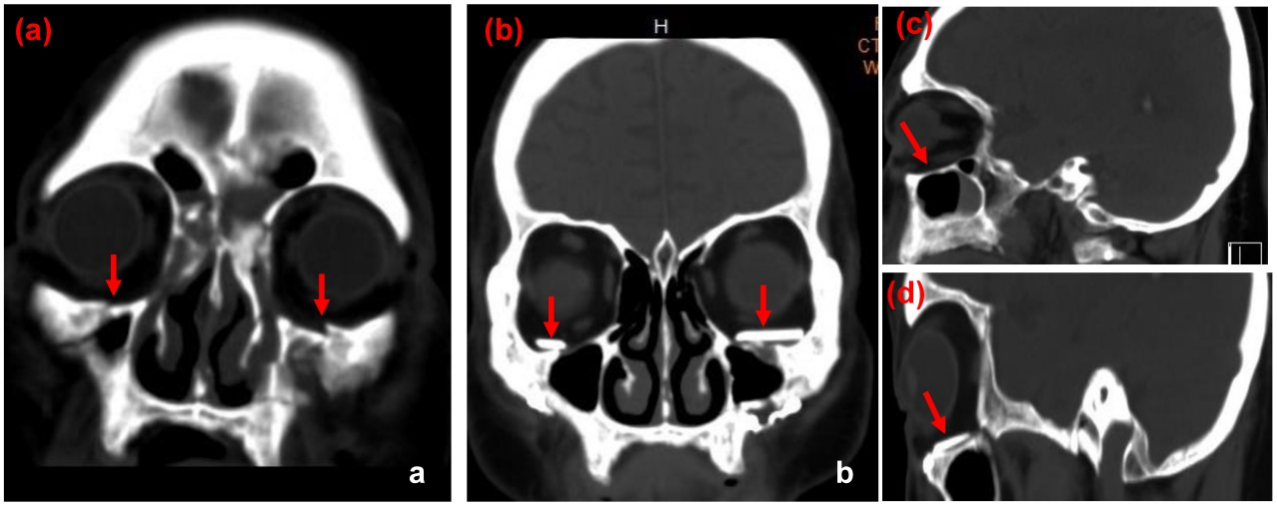
CT images of case 2. The comparison between preoperative CT scan of impaired orbital wall and postoperative CT scan of the orbital wall with ABCcolla® Collagen Bone graft (red arrows). (a) Pre-operative CT image with the coronal view. Fractures of bilateral inferior orbital walls can be seen (red arrows). (b) Post-operative CT image with the coronal view. ABCcolla® Collagen Bone grafts were implanted into bilateral inferior orbital walls (red arrows). (c) Post-operative CT image with the sagittal view. Fractures of bilateral inferior orbital walls are shown (red arrows). (d) Post-operative CT image with the sagittal view. ABCcolla® Collagen Bone graft was implanted into bilateral inferior orbital walls (red arrows).

**Figure 5:**
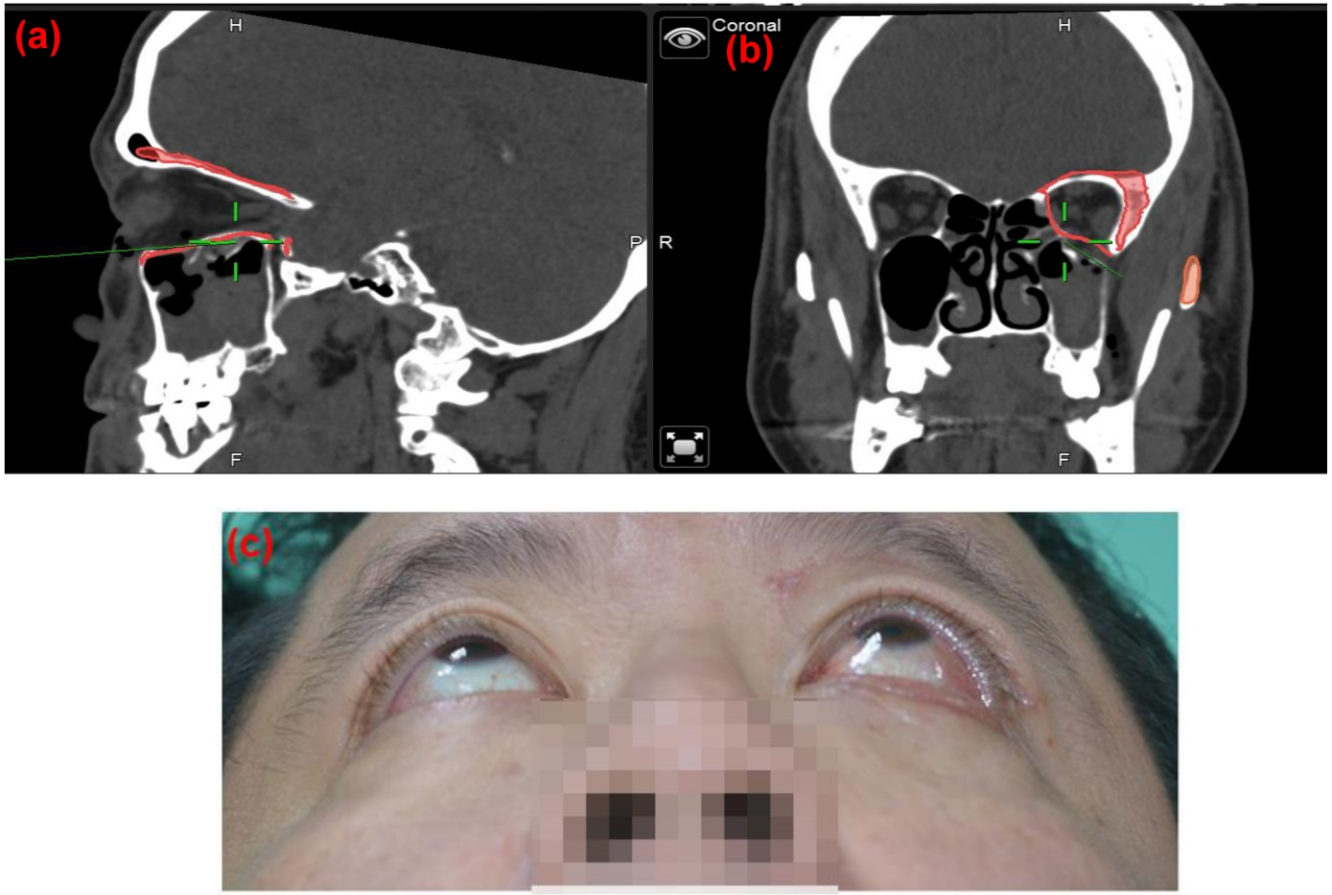
Case 9, 49 y/o male patients, left side orbital floor blowout fracture, Left eye post-op one month, Hertel OD 18mm, OS 21mm,( Left eye exophthalmos 3mm). The navigator assisted surgery allows the surgeon to go into the deep space (green line) which is close to the optic nerve without compromising the optic nerve function.

**Figure 6:**
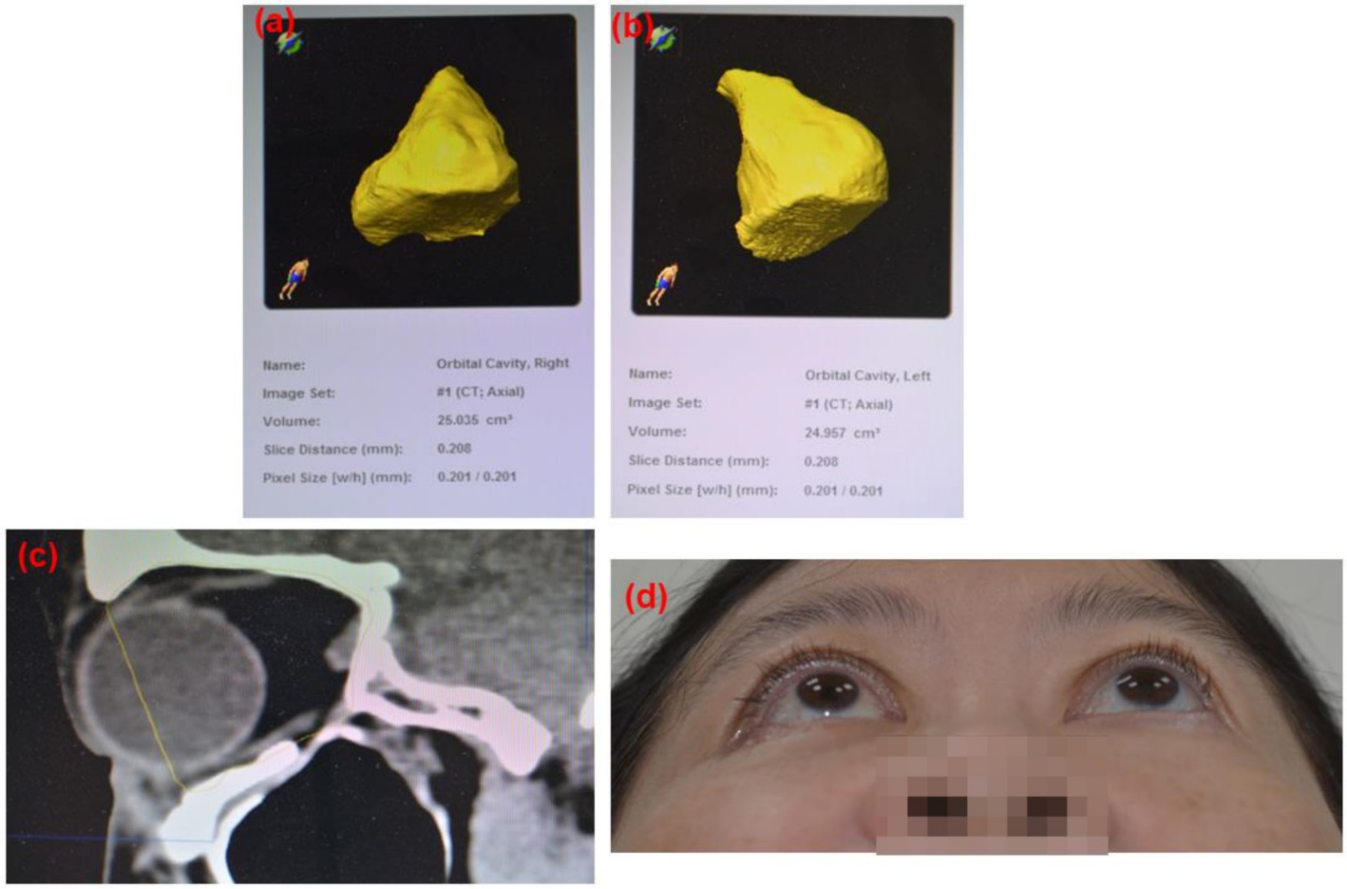
Post-operation 8 weeks follow up CT images based on the 3D reconstruction of the orbital cavity by Brainlab® software of Case 10 (Rt side Zygomatic complex fracture). (a) Rt side orbital volume: 25.035 cm^3^, (b) Lt side orbital volume: 24.957 cm^3^ Difference: 0.078 cm^3^ (c) sagittal view. ABCcolla^®^ Collagen Bone graft was implanted (d) post-operation 2 months follow-up. Without any complaint was noted.

In this present series, the average age of these patients was 50 y/o, ranging from 30 to 65 years old. Among these patients, 2 of them were female and others were males. All the orbital floor fracture sites were reconstructed with customized ABCcolla^®^ Collagen Bone Graft. Each case used one piece of ABCcolla^®^ Collagen Bone Graft except one that used two pieces of ABCcolla^®^ Collagen Bone Graft to reinforce stability in the curved outline of the orbital defect. All of them were arranged follow-ups at the ophthalmologist clinic 4 weeks and 8 weeks after surgery. Brain CT (computed tomography) was taken at the week 8 follow-up visit to evaluate the surgical outcome. These patients showed neither permanent nor significant complications such as limitation of EOM (extraocular movement) postoperatively. The extraocular muscle laceration and periorbital tissue injuries were healed. Pre-operative enophthalmos were notice improving after operation in follow-up (Figures 3-6) and 1 out of 10 cases had residual enophthalmos (Hertel test: 3 mm difference) and one patient had 3 mm exophthalmos (Figure 5) after the operation. None of the 10 cases presented implant migration. No reoperation for implant readjustment was requested. Moreover, neither infection nor immune rejection event was observed in follow-ups.

## Discussion

Ten cases of implantation of ABCcolla^®^ Collagen Bone Graft were successful due to few complications was observed in follow-ups. The ABCcolla^®^ Collagen Bone Graft showed excellent biocompatibility, restoration of ocular symmetry, and strengthened stability. The deviation of orbital alignment in 2 cases can result from surgical limitation to check the posterior orbital wall during the surgery since approaching the posterior orbital wall can damage the optic nerve. The instability of the posterior orbital wall can lead to postoperative graft subsidence.

ABCcolla^®^ Collagen Bone Graft possess a chemical composition similar to that of native bone which is suitable to stimulate osteogenesis 20. The microporosity of the native bone is preserved after the scCO_2_ process in ABCcolla^®^ Collagen Bone Graft. The recent report also indicated the preservation of microarchitecture of the porcine bone derived from a patented decellularization and oxidation process 21. The scCO_2_ process produced ABCcolla^®^ Collagen Bone Graft does not alter the native structure of bone. The various sizes of porous bone structure maintained after scCO_2_ process are essential in angiogenesis and bone growth and bone reorganization in and around the graft material 4, 22.

ABCcolla^®^ Collagen Bone Graft derived from the scCO_2_ extraction technology has been proved to be pyrogen-free. In addition, it did not show any mutagenic effect evaluated by *in vitro* gene mutation analysis in L5178Ytk+/- cells. Systemic toxicity studies have been widely employed to evaluate a medical device's organ toxicity such as in the liver, heart, kidneys, and brain 23. ABCcolla^®^ Collagen Bone Graft showed no evidence of adverse effects, mortality, and noticeable gross lesions in rats.

ABCcolla^®^ Collagen Bone Graft proved good biocompatibility, healing, and bone regeneration in the rabbit osteochondral defects model. The defects created in the distal femoral metaphysis of rabbits were grafted with ABCcolla^®^ Collagen Bone Graft. It performed as an excellent bone substitute that can regenerate the bone void. ABCcolla^®^ Collagen Bone Graft increased new bone formation in void sites, indicating good potential for osteoconductivity 4.

ABCcolla^®^ Collagen Bone Graft is efficient in the regeneration of a critical defect and promoting new bone formation and osteoconduction in rabbit osteochondral defect model. The efficacy of ABCcolla^®^ Collagen Bone Graft on bone regeneration was evaluated in dog mandibular extraction socket in comparison to that of a commercially available Bio‐Oss^®^. The treatment sites with ABCcolla^®^ Collagen Bone Graft revealed a significantly greater stiffness than those of the Bio‐Oss^®^‐treated sites in the biomechanical analysis 4, 22.

Restoration of orbital wall symmetry is critical in recovering the function and aesthetics of the patient's face, therefore multiple approaches have been invented for the orbital wall reconstructions 2. Implants such as titanium mesh, Medpor (porous poly-ethylene) and autologous grafts have been used for orbital reconstruction 6-9, 12. Autologous implants such as calvarial bone, rib, maxillary bone, mandible, and iliac crest have been the standard treatment for orbital wall fracture 3, 7, 9. Though autologous grafts tend to have low infection rates and rare immune rejection 3, 9, the morbidity of the donor sites, inelastic nature of the materials and varied reabsorption rate are unneglectable, leading to the invention of other materials such as titanium mesh 3, 9.

Titanium mesh, a metallic alloplastic material, plays an important role in the fixation of the large defect (>2 cm) due to its high resistance 10. It also displays great osteointegration 2. The study by Schubert et al. in 2002 on 8 patients showed great biocompatibility of titanium mesh with soft tissue 9, 24. Woo et al. in 2014 on 17 patients also demonstrated a satisfactory outcome in the use of titanium mesh 25. Mackenzie et al. reported only 1 out of 51 cases of orbital reconstruction showed enophthalmos after 9 months follow-ups 26.

To increase the flexibility of titanium mesh, Medpor^®^ (porous polyethylene), integrating both titanium mesh and high-density polyethylene sheets, was invented. Medpor^®^ is a porous framework that facilitates fibrovascular ingrowth and tissue integration 3, 8, 11. The flexibility can accurately bridge orbital defects, restoring precise symmetry of orbital walls 3, 8, 11. It also possesses the advantages such as low infection rate and low foreign body rejection rate 8, 11. A retrospective study done by Garibaldi et al. reviewed the clinical outcomes of 106 patients who underwent orbital reconstruction with Medpor^®^ implantation 12. 7 out of 106 presented complications such as retrobulbar haemorrhage, transient oculomotor disturbance and vertical overcorrection. None of these cases presented implant extrusions or infection after surgery 12. These results favoured Medpor^®^ as excellent support of the orbital wall. However, Medpor^®^ cannot be seen on a radiograph, making it hard to check post-operative position on X-ray 3. In addition, the cost of Medpor^®^ can be a drawback, it will be a burden on financially disadvantaged patients.

Up until this stage, there is no definite choice of materials in terms of orbital wall reconstruction. Thus, exploration of novel alternatives such as the xenograft framework can be a solution to this issue 27. Xenograft decellular framework such as ABCcolla^®^ Collagen Bone Graft has been gaining popularity in orthopedic and dental applications, but its application in orbital wall reconstruction was not yet explored 4. The result of this study favoured ABCcolla^®^ Collagen Bone Graft as a strong implant covering over the orbital defect with low complication rate, low infection rate, low cost, high biocompatibility and high osteoconductive properties. To further explore long-term possible complications of ABCcolla^®^ Collagen Bone Graft, more clinical studies are to be done in the future. In addition, long-term follow-ups of the existing cases were also critical to further evaluate bone remodeling, regeneration, and reabsorption. However, preclinical studies depicted ABCcolla® Collagen Bone Graft undergone bone remodeling, regeneration, and reabsorption 22. As the first clinical study on orbital wall reconstruction with ABCcolla^®^ Collagen Bone Graft implantation, it shed light on the potential application of the xenograft de-cellular framework to regain functions of orbital walls.

## Conclusions

Reconstruction of the orbital floor fracture has been a challenge in plastic surgery due to the delicacy and complexity of bone arrangements in the orbital cavity. Numerous types of implants have been explored such as autografts, allogenic materials and alloplastic materials (8). Since each kind of material has unique properties, there is no conclusive treatment plan for orbital wall reconstruction. Therefore, this study provides evidence and proves that the xenograft decellular framework, ABCcolla^®^ Collagen Bone Graft is a new alternative to autografts, allogenic materials and alloplastic materials for orbital floor reconstruction. The result of this study proved ABCcolla^®^ Collagen Bone Graft is a suitable graft material with optimal clinical outcomes in orbital floor reconstruction.

## Figures and Tables

**Table 1:** Patient information

Patient	No.1	No.2	No.3	No.4	No.5	No.6	No.7	No.8	No.9	No.10
sex	Female	Male	Male	Male	Male	Male	Male	Male	Male	female
Age	30	65	64	58	50	48	35	45	49	56
Cause of injury	Traffic accident	Traffic accident	Traffic accident	Traffic accident	falling	falling	Traffic accident	Conscious disturbance	Traffic accident	Traffic accident
Hertel test*	**n/a**	**OD:13, OS:10**	**n/a**	**n/a**	**n/a**	**n/a**	**n/a**	**n/a**	**OD: 18, OS: 21**	**n/a**

OD: right eye; OS: left eye. * Hertel test was performed only when enophthalmous was noticed by ophthalmologists in follow-ups.

**Table 2:** Comparison of 4 different materials for orbital wall reconstruction

Materials	autologous grafts	Titanium mesh	Medpor® (porous polyethylene)	ABCcolla® Collagen Matrix
Donor site morbidity	---	-	-	-
Infection resistance	++	+	+	+
biocompatible	++	+	+	+
Stiffness	++	++	+	++
Visible on image	+	+	---	+
Cost-effective	++	+	+	++

---Poor, - not applicable, + fair, ++ good.

## References

[1] Heo JJ, Chong JH, Han JJ (2018). Reconstruction of the orbital wall using superior orbital rim osteotomy in a patient with a superior orbital wall fracture. Maxillofac. Plast. Reconstr. Surg.

[2] Ellis E 3rd, Tan Y (2003). Assessment of internal orbital reconstructions for pure blowout fractures: cranial bone grafts versus titanium mesh. J. Oral Maxillofac. Surg.

[3] Potter JK, Malmquist M, Ellis E 3rd (2012). Biomaterials for the reconstruction of the internal orbit. Oral Maxillofac. Surg. Clin. North Am.

[4] Chen YW, Hsieh DJ, Periasamy S, Yen KC, Wang HC, Chien HH (2021). Development of a decellularized porcine bone graft by supercritical carbon dioxide extraction technology for bone regeneration. J. Tissue Eng. Regen. Med.

[5] Chou C, Kuo YR, Chen CC, Lai CS, Lin SD, Huang SH, Lee SS (2017). Medial Orbital Wall Reconstruction With Porous Polyethylene by Using a Transconjunctival Approach With a Caruncular Extension. Ann. Plast. Surg.

[6] Kim YH, Park Y, Chung KJ (2016). Considerations for the Management of Medial Orbital Wall Blowout Fracture. Arch. Plast. Surg.

[7] Lozada KN, Cleveland PW, Smith JE (2019). Orbital Trauma. Semin. Plast. Surg.

[8] Iordanidou V, De Potter P (2004). Porous polyethylene orbital implant in the pediatric population. Am. J. Ophthalmol.

[9] Mok D, Lessard L, Cordoba C, Harris PG, Nikolis A (2004). A review of materials currently used in orbital floor reconstruction. Can. J. Plast. Surg.

[10] Gear AJ, Lokeh A, Aldridge JH, Migliori MR, Benjamin CI, Schubert W (2002). Safety of titanium mesh for orbital reconstruction. Ann. Plast. Surg.

[11] Lin IC, Liao SL, Lin L (2007). Porous Polyethylene Implants in Orbital Floor Reconstruction. J. Formos. Med. Assoc.

[12] Garibaldi DC, Iliff NT, Grant MP, Merbs SL (2007). Use of porous polyethylene with embedded titanium in orbital reconstruction: a review of 106 patients. Ophthalmic Plast. Reconstr. Surg.

[13] Chou PR, Lin YN, Wu SH, Lin SD, Srinivasan P, Hsieh DJ, Huang SH (2020). Supercritical Carbon Dioxide-decellularized Porcine Acellular Dermal Matrix combined with Autologous Adipose-derived Stem Cells: Its Role in Accelerated Diabetic Wound Healing. Int. J. Med. Sci.

[14] Lee SS, Wu YC, Huang SH, Chen YC, Srinivasan P, Hsieh DJ, Yeh YC, Lai YP, Lin YN (2021). A novel 3D histotypic cartilage construct engineered by supercritical carbon dioxide decellularized porcine nasal cartilage graft and chondrocytes exhibited chondrogenic capability in vitro. Int. J. Med. Sci.

[15] Huang YH, Tseng FW, Chang WH, Peng IC, Hsieh DJ, Wu SW, Yeh ML (2017). Preparation of acellular scaffold for corneal tissue engineering by supercritical carbon dioxide extraction technology. Acta Biomater.

[16] Fages J, Marty A, Delga C, Condoret JS, Combes D, Frayssinet P (1994). Use of supercritical CO2 for bone delipidation. Biomaterials.

[17] You L, Weikang X, Lifeng Y, Changyan L, Yongliang L, Xiaohui W, Bin X() (2018). In vivo immunogenicity of bovine bone removed by a novel decellularization protocol based on supercritical carbon dioxide. Artificial Cells, Nanomedicine and Biotechnology.

[18] Susarla SM, Duncan K, Mahoney NR, Merbs SL, Grant MP (2015). Virtual surgical planning for orbital reconstruction. Middle East Afr. J. Ophthalmol.

[19] Bronstein JA, Bruce WJ, Bakhos F, Ishaq D, Joyce CJ, Cimino V (2020). Surgical approach to orbital floor fractures: comparing complication rates between subciliary and subconjunctival approaches. Craniomaxillofac. Trauma Reconstr.

[20] Maté Sánchez de Val JE, Calvo-Guirado JL, Gómez-Moreno G (2016). Pérez-Albacete Martínez C, Mazón P, De Aza PN. Influence of hydroxyapatite granule size, porosity, and crystallinity on tissue reaction in vivo. Part A: Synthesis, characterization of the materials, and SEM analysis. Clin. Implant Dent. Relat. Res.

[21] Bracey DN, Seyler TM, Jinnah AH, Lively MO, Willey JS, Smith TL, Whitlock PW (2018). A decellularized porcine xenograft derived bone scaffold for clinical use as a bone graft substitute: A critical evaluation of processing and structure. J Funct Biomater.

[22] Chen YW, Chen MY, Hsieh DJ, Periasamy S, Yen KC, Chuang CT, Wang HC, Tseng FW, Kuo JC, Chien HH (2020). Evaluating the bone-regenerative role of the decellularized porcine bone xenograft in a canine extraction socket model. Clin Exp Dent Res.

[23] International Organisation for Standardisation [ISO]. (2017). ISO 10993- 11: Biological evaluation of medical devices - Part 11 tests for systemic toxicity.

[24] Schubert W, Gear AJ, Lee C, Hilger PA, Haus E, Migliori MR, Mann DA, Benjamin CI (2002). Incorporation of titanium mesh in orbital and midface reconstruction. Plast. Reconstr. Surg.

[25] Woo KS, Cho PD, Lee SH (2014). Reconstruction of severe medial orbital wall fractures using titanium mesh plates by the pericaruncular approach. J. Plast. Surg. Hand Surg.

[26] Mackenzie DJ, Arora B, Hansen J (1999). Orbital floor repair with titanium mesh screen. J. Craniomaxillofac. Trauma.

[27] Amini Z, Lari R (2021). A systematic review of decellularized allograft and xenograft-derived scaffolds in bone tissue regeneration. Tissue Cell.

